# Supplementary material for: Comparison of intravenous magnesium sulphate and lidocaine for attenuation of cardiovascular response to laryngoscopy and endotracheal intubation in elective surgical patients at Zewditu Memorial Hospital Addis Ababa, Ethiopia
Source: PLoS One. 2021 Jun 1;16(6):e0252465. doi: 10.1371/journal.pone.0252465 (PMC8168879; doi:10.1371/journal.pone.0252465)
Supplement: S2 File — (DOCX) [file pone.0252465.s002.docx]

### Annex: IV Data collection tool

**Date ____/___/____**

**Check list Code _____________**

Data collection tool (questionnaire) for patient who will have taken general anesthesia induced by thiopental, premedicated either by lidocaine or magnesium sulphate at zewditu memorial hospital November 2018.

Instructions:

A. Fill the blank space provided.

B. Encircle the alternatives when necessary.

**Part I**: Questions on socio-demographic and physical characteristics of the patient

| 101 | Age (years ) | ------------------------- years |
| --- | --- | --- |
| 102 | Sex | A, Male , B, Female |
| 103 | Weight in Kg | ………………………. |
| 104 | Height in meter | …………………….. |
| 105 | Diagnosis | ……………………….. |
| 106 | BMI | ………….. |
| 107 | ASA physical status | A, ASA I B, ASA II |
| 108 | Is there any co existing medical disease? | A, Yes B, No |
| 109 | If yes; specify the disease | ------------ |

**Part II**: Questions about anesthetic characteristics of the patient.

| 201 | The used anesthetic adjuvant five minutes before intubation | A, Magnesium sulphate(Mgso4)(……….mg)  B, Lidocaine(………………….mg)  C, Induction without lidocaine or Mgso4  D, Tramodol…………….mg  E, other (…………………...) |
| --- | --- | --- |
| 202 | DOSE of induction agent(thiopental) | ……. |
| 203 | Intubating muscle relaxant | A, suxamethonium  B, vecronium  C, pancronium  D, specify any others……………………… |
| 204 | Number of attempts to intubate | A, 1  B, 2 |

**Part III perioperative hemodynamic parameter measurements.**

**3, Hemodynamic parameter**

| Hemodynamic parameter | Heart rate | Systolic blood pressure | Diastolic blood pressure | Mean arterial blood pressure | Spo2 |
| --- | --- | --- | --- | --- | --- |
| 301, Before injection of lidocaine or Mgso4 if used. Or before thiopental injection if no lidocaine or Mgso4( base line ) |  |  |  |  |  |
| 302**,** 1 minute after induction of anesthesia |  |  |  |  |  |
| 303, immediately after intubation |  |  |  |  |  |
| 304, 2 minutes after intubation |  |  |  |  |  |
| 305, 5 minutes after intubation |  |  |  |  |  |
| 306, 7 minutes after intubation |  |  |  |  |  |

**4, Maintenance of muscle relaxant and inhalational agent opened immediately after intubation**

| 401, Halothane | A, 1 % B,1.5 % C, 2%  D, specify any other MAC…………. |
| --- | --- |
| 402, Isoflurane | A, 1 % B,1.5 % C, 2%  D, specify any other MAC…………. |
| 403, muscle relaxant within 7 minutes of intubation. If there specify minutes …………. | A, suxamethonium C,pancronium  B, vecronium D,atracurium  E, no relaxant within 7 minutes |
| 404, Is surgery started within 7 minutes of intubation? | A yes, B no; if yes specify minutes………………. |
| 405, Is opioid given within 7 minutes of intubation?  Specify…………….. | A yes, B no; if yes specify minutes………………. |

# Appendix one

**Classification of Obesity**

| BMI(kg/m2) | Description |
| --- | --- |
| <18.5 | Underweight |
| 18.5–24.9 | Normal |
| 25–29.9 | Overweight |
| 30–34.9 | Obesity (class I) |
| 35–39.9 | Obesity (class II) |
| ≥40 | Morbid obesity (class III) |
| ≥50 | Super obesity |
| ≥60 | Super -super obesity |

Adopted from Paul G. Barash clinical anesthesia 7^th^ edition.

**American Society of Anesthesiologists (ASA) physical status classification of patients.**

| **Class** | **Definition** |
| --- | --- |
| 1 | Normal healthy patient |
| 2 | Patient with mild systemic disease (no functional limitations) |
| 3 | Patient with severe systemic disease (some functional limitations) |
| 4 | Patient with severe systemic disease that is a constant threat to life (functionality incapacitated) |
| 5 | Moribund patient who is not expected to survive without the operation |
| 6 | Brain-dead patient whose organs are being removed for donor purposes |
| E | If the procedure is an emergency, the physical status is followed by “E” (for example, “2E”) |

Adopted from Morgan and Mikhail 5^th^ edition
