# Supplementary material for: Comparison of intravenous magnesium sulphate and lidocaine for attenuation of cardiovascular response to laryngoscopy and endotracheal intubation in elective surgical patients at Zewditu Memorial Hospital Addis Ababa, Ethiopia
Source: PLoS One. 2021 Jun 1;16(6):e0252465. doi: 10.1371/journal.pone.0252465 (PMC8168879; doi:10.1371/journal.pone.0252465)
Supplement: Attachment — Submitted filename: response for reviewers.docx [file pone.0252465.s003.docx]

First of all we would like to say thank you all reviewers for their detailed review. Then let us clear some questions and doubts raised by reviewers

Reviewer 1 (Hans Bahlmann)

1. Why only every second patient was chosen? We used propoablity sampling method (SRS) and the calculation based on systematic random sampling is every second patient.
2. P9. Group allocation is based on anesthetist’s preference? Our data collectors observed and collected data which fulfils the inclusion criteria then if the required number is found in any group before study period is finished they only collected the remain group. E.g. In Lidocaine group the required number reached within 3 months.
3. P12. Table 3 contains p value of between the group and within the group p values. So at 5 min the p value between Group M and Group L is 0.029. and the p value among groups was <0.001
4. P22 our conclusion is based on our finding since the post-hoc analysis shows there is a statistically significance difference between lidocaine and magnesium groups.

Even though the nature of a study design unable to control confounding factors like vaporizers, intubation skill we exclude difficult intubations in the study. And there was no difference among the groups in the type and MAC of inhalational agents.

Minor comment

1. P5. ‘Please describe which difference was deemed significant for the sample size calculation ‘? The Iranian study compared HR, SBP, DBP and MAP. We checked the difference between groups for all parameters and the difference was largest in MAP so we used this one for our sample calculation.
2. P7. Why opioid like fentanyl was not used? In Ethiopia fentanyl was not available in the market and in study setting anesthetists used tramadol.
3. Drug dose was based on actual body weight.
4. Both lidocaine and magnesium were injected before 5 minutes of induction of anesthesia.
5. Table 1 MAC at what time point was MAC assessed? Since the study minutes was short (within 7 minutes) there was no great dynamics and we took the average value within 7 minutes of intubation.

Reviewer2

1. Does cardiovascular reflex always happen in all setting? It may not necessarily in all setting but it happen at least in majority cases.
2. What type of cardiovascular response do you expect? The common ones are tachycardia, hypertension and arrhythmia are common responses to laryngoscopic stimulation.
3. How do you give similar care for study subjects and who refused to participate? We just observed the event and if participants refused we only exclude from study participants otherwise the hospital anesthetists gave similar care for those included and excluded patients. Care was independent of being participant or not.
4. Yes we agreed it is better if it was studied in RCT design, and how you can compare drugs in observational study? Even though it has limitation the hospital practice is somehow favorable for this study design from our previous observation and we tried our best to compare drugs with this study design.

Reviewer 3(Nora Terrasini;MD, PHD)

1. Lidocaine and magnesium sulphate were given before 5 minutes of induction of anesthesia. Both drugs were given in IV push and magnesium sulphate was injected slowly within 5 minutes. After 5 minutes of pretreatment anesthetists induced anesthesia.
