# Supplementary material for: Comparison of intravenous magnesium sulphate and lidocaine for attenuation of cardiovascular response to laryngoscopy and endotracheal intubation in elective surgical patients at Zewditu Memorial Hospital Addis Ababa, Ethiopia
Source: PLoS One. 2021 Jun 1;16(6):e0252465. doi: 10.1371/journal.pone.0252465 (PMC8168879; doi:10.1371/journal.pone.0252465)
Supplement: Attachment — Submitted filename: response for reviewers.docx [file pone.0252465.s004.docx]

First of all, we would like to acknowledge all reviewers for giving your time to review our manuscript and response letter. Then our responses for reviewers’ comment are put as follow.

Reviewer 1

Dear reviewer!; we accept your concern regarding confounding factors, but we were thinking in observational studies these confounding factors are default and that is why we did not mention. Now we have incorporated the limitation and relevance of the study in the manuscript.

The other concern is ‘the choice of the study drug is by the preference of anesthetist’. In this point what we want to say is the nature of the study is observational and we did not enforce anesthetists to change their plan for the purpose of our study. The data collectors just observe what was happening and collecting data. The study was done in resource limited country, so with it’s limitations we think this study can give information for researchers. Therefore, dear reviewer please review our manuscript for the third time thank you.

Reviewer2

Dear reviewer! Thank you for assuring as we have addressed your comments

Reviewer 3

Dear reviewer! we have tried to rewrite the manuscript with language experts
