# Supplementary material for: Comparison of intravenous magnesium sulphate and lidocaine for attenuation of cardiovascular response to laryngoscopy and endotracheal intubation in elective surgical patients at Zewditu Memorial Hospital Addis Ababa, Ethiopia
Source: PLoS One. 2021 Jun 1;16(6):e0252465. doi: 10.1371/journal.pone.0252465 (PMC8168879; doi:10.1371/journal.pone.0252465)
Supplement: Attachment — Submitted filename: response to reviewers.docx [file pone.0252465.s005.docx]

First of all, we would like to acknowledge all reviewers for giving your time to review our manuscript repeatedly. Then we have tried to reflect our responses as follow for reviewers according to concerns.

Reviewer 1

Dear reviewer! Thank you for your concern to be our article scientifically sounded. And you are not satisfied on our conclusion ‘magnesium is better than lidocaine’. Even though we concluded standing from the statistical analysis we can accept your expression ‘pretreatment of magnesium sulphate is associated with a more favorable hemodynamic response’. For our study design we all are comfortable for this expression and will take it as it is. The other concern is ‘the choice of the study drug is by the preference of anesthetist’. The pushing factor to study on this topic is our clinical observation, anesthetists in our study setting are using these drugs as a prophylaxis but there is a confusion which agent is more preferable than the other. So based on this situation we designed observational cohort to study this topic and we are not sure whether it is limitation or not. But you strongly argued this is the limitation of the study so we have incorporated in the manuscript.

Reviewer 3

Dear reviewer! Thank you for assuring as we have addressed your comments
