# Supplementary material for: Comparison of intravenous magnesium sulphate and lidocaine for attenuation of cardiovascular response to laryngoscopy and endotracheal intubation in elective surgical patients at Zewditu Memorial Hospital Addis Ababa, Ethiopia
Source: PLoS One. 2021 Jun 1;16(6):e0252465. doi: 10.1371/journal.pone.0252465 (PMC8168879; doi:10.1371/journal.pone.0252465)
Supplement: Attachment — Submitted filename: Response to Reviewers.docx [file pone.0252465.s006.docx]

First of all, we would like to acknowledge reviewer1 for giving your time to review our manuscript repeatedly. Then we corrected the manuscript

Reviewer 1

Dear reviewer! Thank you so much! For reviewing each components of the manuscript carefully. And we have corrected it.
